# Predictive Ability of Plasma p‐tau217 for β‐Amyloid Status: A Prospective Multicenter Study

**DOI:** 10.1002/acn3.70387

**Published:** 2026-04-13

**Authors:** Miquel Massons, Nuria Guillen, Jordi Sarto, Neus Falgàs, Sergi Borrego‐Écija, Diana Esteller‐Gauxax, Magda Castellví, Adrià Tort‐Merino, Agnès Pérez‐Millan, Anna Antonell, Josep M. Augè Fradera, Gerard Piñol, Iolanda Riba, Anna Carnes‐Vendrell, Marta Cullell, Maria Teresa Osuna, Lorena Bajo, Teresa Romero, Eva Bonjoch, Joan Bello, Susana Fernández, Marta Balagué, Isabel Gómez‐Ruiz, Anuncia Boltes, Claustre Pont, Raquel Cuevas, Sara Carrillo, Laura Iglesias, Teresa Maria Casadevall Codina, Lorena Grau Guinea, Fernando Jose Espada, Raquel Sánchez‐Valle, Mircea Balasa, Albert Lladó

**Affiliations:** ^1^ Alzheimer's Disease and Other Cognitive Disorders Unit, Hospital Clínic de Barcelona, Fundació de Recerca Clínic Barcelona – Institut d'Investigacions Biomèdiques August Pi i Sunyer (IDIBAPS) Barcelona Spain; ^2^ University of Barcelona Barcelona Spain; ^3^ Centro de Investigación Biomédica en Red en Enfermedades Neurodegenerativas (CIBERNED) Madrid Spain; ^4^ Hospital Universitari Santa Maria Gestió Serveis Sanitaris Lleida Spain; ^5^ eHealth Center, Faculty of Computer Science, Multimedia and Telecommunications, Universitat Oberta de Catalunya Barcelona Spain; ^6^ Hospital de Figueres Fundació Salut Empordà Figueres Spain; ^7^ Fundació Hospital de la Santa Creu de Vic Vic Spain; ^8^ Hospital Moisés Broggi‐Consorci Sanitari Integral Sant Joan Despí Spain; ^9^ Fundació Privada Hospital Asil de Granollers Granollers Spain; ^10^ Hospital Sant Jaume de Calella Corporació de Salut del Maresme i la Selva Calella Spain

**Keywords:** Alzheimer's disease, blood‐based biomarkers, multicenter study, plasma, p‐tau217

## Abstract

**Objective:**

Plasma tau phosphorylated at threonine 217 (p‐tau217) measured with fully automated platforms has shown high accuracy for Alzheimer's disease (AD) diagnosis, but real‐world multicenter data remain limited. We aimed to validate the diagnostic performance of p‐tau217 for identifying AD pathology in a real‐world multicenter cohort across seven memory clinics in Catalonia (Spain), with only one tertiary hospital with prior experience in AD blood‐based biomarkers.

**Methods:**

In this prospective multicenter study, consecutive patients with cognitive impairment undergoing routine cerebrospinal fluid (CSF) biomarker testing were included. Plasma samples were collected following a standardized pre‐analytical protocol and analyzed centrally using the Lumipulse G p‐tau217 assay (Fujirebio). Diagnostic accuracy for Aβ status was assessed overall and across sites.

**Results:**

A total of 185 participants were included. Plasma p‐tau217 showed excellent accuracy for CSF‐defined Aβ status (AUC 0.916) with consistent performance across centers. Using a single cut‐off, diagnostic accuracy reached 84.9%, which prompted the use of a dual‐threshold strategy to improve overall performance and to classify p‐tau217 values into low, intermediate, and high probability categories of Aβ positivity. When applying a strict model with 97.5% sensitivity and specificity (cut‐offs 0.146/0.486 pg/mL), 42.7% of participants fell within the intermediate zone, whereas the remaining 57.3% were confidently classified with 95.3% accuracy.

**Interpretation:**

In a real‐world multicenter memory‐clinic cohort, plasma p‐tau217 measured on a fully automated platform accurately discriminated CSF Aβ status and enabled reliable rule‐in/rule‐out classification in over half of patients. These findings support its broader clinical use as an initial diagnostic tool for AD.

## 1 Introduction

Alzheimer's disease (AD) is the leading cause of dementia and requires timely, accurate diagnosis to guide care planning, enable access to disease‐modifying therapies, and support clinical trial enrollment [1, 2, 3]. Contemporary diagnostic frameworks define AD biologically using cerebrospinal fluid (CSF) and positron emission tomography (PET) biomarkers that capture amyloidosis (A or Aβ; CSF Aβ42/Aβ40 or amyloid‐PET) and tauopathy (T; CSF phosphorylated tau or tau‐PET) [4, 5, 6]. However, the cost, invasiveness, and limited availability of CSF/PET restrict their use; consequently, only a minority of patients undergo biomarker testing, and diagnostic misclassification remains substantial—in primary care and even in specialty memory clinics [7, 8, 9, 10].

Blood‐based biomarkers (BBMs) have emerged as minimally invasive, scalable alternatives that can even replace or triage patients for confirmatory CSF/PET testing. Among them, plasma phosphorylated tau species closely track AD biology and outperform Aβ42/40, neurofilament light chain (NfL), and glial fibrillary acidic protein (GFAP) for identifying CSF/PET‐defined amyloidosis; in particular, p‐tau217 performs excellently and is closely associated with both Aβ and T [11, 12, 13, 14, 15, 16, 17, 18, 19]. Reflecting this progress, revised criteria contemplate BBM‐only diagnostic pathways when performance exceeds 90% accuracy [20]. Fully automated immunoassay platforms further enhance scalability and standardization, mirroring prior CSF experience and growing evidence in plasma workflows [21, 22]. Head‐to‐head and cross‐platform studies—including comparisons with mass spectrometry—show high accuracy for plasma p‐tau217 and support its clinical utility across settings [16, 17, 18, 19, 23, 24, 25].

Adding to this evidence base, we recently demonstrated in a consecutive cohort (Hospital Clínic de Barcelona (HCB); *n* = 468; 2019–2024) that plasma p‐tau217 measured by Lumipulse outperformed p‐tau181 measured on Simoa for discriminating CSF Aβ status (AUC 0.95 vs. 0.90; *p* = 0.005). Using a dual‐threshold approach targeting 97.5% sensitivity/specificity, 67% of patients were confidently classified into low/high groups with 96% accuracy [26].

Despite these promising results, most available evidence derives from single‐center or research‐enriched cohorts, limiting generalizability. Multicenter evaluations are therefore needed to assess between‐site reproducibility on commercial automated platforms and to generate evidence grounded in routine clinical practice [9, 27]. Implementing dual‐threshold strategies (low/intermediate/high) can further improve applicability by sparing confirmatory CSF/PET in clear‐cut cases and appropriately routing indeterminate results, thereby enhancing access and equity [25].

We now present a prospective multicenter study conducted across seven memory clinics in Catalonia, Spain. The primary aim of this study was (1) to validate the diagnostic performance of plasma p‐tau217 measured with Lumipulse for identifying AD pathology, as defined by CSF Aβ42 or Aβ 42/40 ratio, across a multicenter cohort, including both tertiary and non‐tertiary sites. Secondary objectives were (2) to examine the relationship between CSF A/T status and plasma p‐tau217 values, and (3) to assess the influence of demographic and clinical factors on diagnostic accuracy.

## 2 Methods

### 2.1 Design

A prospective multicenter study evaluating the diagnostic performance of plasma p‐tau217 for the detection of brain amyloid status, as defined by the CSF Aβ42 or Aβ 42/40 ratio.

### 2.2 Study Population

Individuals with cognitive impairment consecutively referred to seven memory clinics in Catalonia (Spain) between June 2023 and February 2024 were prospectively included. The participating centers were: Hospital Clínic de Barcelona (HCB), Hospital Universitari Santa Maria de Lleida, Hospital de Figueres, Hospital de la Santa Creu de Vic, Hospital Moisés Broggi–Consorci Sanitari Integral, Hospital Asil de Granollers, and Hospital Sant Jaume de Calella. Among them, HCB was the only tertiary hospital with previous experience in the validation and clinical implementation of plasma biomarkers for AD diagnosis.

The inclusion criteria were the presence of cognitive impairment in the clinical stage of mild cognitive impairment (MCI) or mild dementia, and available results of CSF AD biomarkers obtained as part of the routine diagnostic work‐up. Final diagnostic classification was performed according to established international diagnostic criteria for ad [4, 5, 6], dementia with Lewy bodies (DLB) [28], frontotemporal dementia (FTD) as an umbrella term, including all the clinical variants associated with frontotemporal lobar degeneration (language, behavioral, progressive supranuclear palsy and corticobasal syndrome phenotypes) [29, 30, 31, 32], or suspected non‐neurodegenerative cognitive impairment (SND). SND was defined as a stable cognitive impairment that was not suggestive of any neurodegenerative disease and with normal/negative CSF AD biomarkers.

### 2.3 Procedures and Measurements

#### 2.3.1 Clinical Assessment

Demographic, clinical, and laboratory data, as well as CSF AD biomarkers, were prospectively collected as part of the routine clinical assessment at the participating centers. Clinical assessments were performed by neurologists or geriatricians and included detailed medical history, physical and neurological examinations, comprehensive neuropsychological testing, and structural neuroimaging.

#### 2.3.2 CSF Biomarkers

Lumbar punctures for cerebrospinal fluid (CSF) collection were performed according to current clinical guidelines. CSF amyloid β1–42 (Aβ42), amyloid β1–40 (Aβ40), total tau (t‐tau), and phosphorylated tau at threonine 181 (p‐tau181) were quantified using fully automated chemiluminescent enzyme immunoassays (CLEIA) on the Lumipulse platform (Fujirebio) across all participating centers. Amyloid‐positive (Aβ+) and tau‐positive (T+) status were defined according to site‐specific cut‐offs, on the basis of previously validated center‐specific thresholds (Supplementary Table 1). Following the routine diagnostic workflow of each center, at HCB and two other sites, CSF Aβ42 was used as the primary marker of amyloid status in cases where its values were concordant with CSF phosphorylated tau and CSF total tau (i.e., all three normal or all three pathological), whereas the CSF Aβ42/Aβ40 ratio was used for the rest of the cases. In the remaining centers, amyloid status was determined directly through the CSF Aβ42/Aβ40 ratio.

#### 2.3.3 Blood Biomarkers

Blood samples for p‐tau217 determination were collected at enrollment following a standardized protocol. Samples were centrifuged at 1800 × g for 10 min at room temperature within 2 h of collection to separate plasma, which was then aliquoted into polypropylene tubes (500 μL) and stored immediately at −80°C until analysis. Plasma samples were analyzed in a centralized laboratory at the Service of Biochemistry and Molecular Genetics, HCB. Samples from the six external centers were shipped to HCB at the conclusion of the enrollment period under frozen conditions. Concentrations of p‐tau217 were measured by CLEIA using the Lumipulse G pTau217 kit (Fujirebio), following the manufacturer's protocols and international recommendations [33].

### 2.4 Statistical Analysis

Continuous demographic, clinical, and biomarker variables were summarized as mean ± standard deviation (SD). Normality was evaluated with the Shapiro–Wilk test. Group differences by Aβ status and by recruitment center were assessed using Student's *t*‐test or the Mann–Whitney *U* test for continuous variables, and *χ* ^2^ test for categorical variables.

The diagnostic performance of plasma p‐tau217 for identifying CSF‐defined Aβ positivity was evaluated using receiver operating characteristic (ROC) analyses, with areas under the curve (AUCs) compared using DeLong's method. A cohort‐level optimal cut‐off was derived using the Youden index. To assess between‐site consistency, diagnostic performance was compared between the tertiary hospital and the other six grouped centers. This approach was selected because the tertiary site was the only center with prior experience using p‐tau217 and also served as the analytical laboratory, whereas the remaining centers were implementing the assay for the first time.

To translate plasma concentrations into clinically actionable categories, two predefined dual‐threshold strategies (lenient and strict; targeting 95% and 97.5% sensitivity/specificity, respectively) were applied to classify individuals as having a low, intermediate, or high probability of Aβ positivity. Associations between plasma p‐tau217 and CSF AT biomarker profiles were examined using the Kruskal–Wallis test followed by Dunn's post hoc comparisons.

To explore whether demographic and clinical variables influenced plasma p‐tau217 concentrations, we fitted linear regression models adjusted for age, sex, and CSF‐defined Aβ status. Continuous covariates (age, BMI, and eGFR) were transformed to z‐scores to obtain standardized β coefficients with multivariate linear regression models. Then, to determine whether these variables affected the diagnostic performance, logistic regression models were fitted, including plasma p‐tau217 alone or in combination with BMI and/or eGFR. All analyses were conducted in R (https://www.r‐project.org) (v4.5.0). Two‐sided *p*‐values < 0.05 were considered statistically significant.

### 2.5 Ethical Considerations

This study was conducted in accordance with the Declaration of Helsinki and applicable Spanish regulations governing research with human subjects. The study was approved by the Ethics Committees of all participating centers, including the Hospital Clínic de Barcelona (HCB/2023/0574). All participants provided written informed consent for clinical data and biomarker collection.

## 3 Results

### 3.1 Demographic and Clinical Characteristics

A total of 212 participants were recruited. Of these, 27 were excluded from the final analysis: one due to insufficient plasma sample volume, and 26 because of extreme outlier biomarker values most likely attributable to pre‐analytical issues. Specifically, 21 of 23 participants from one center showed uniformly elevated plasma p‐tau217 concentrations inconsistent with the rest of the cohort (range 0.931–7.216 pg/mL). Although the remaining two participants from that center had seemingly plausible concentrations (0.204 and 0.519 pg/mL), they were also excluded as a precaution given the site‐wide anomaly. In addition, three participants from two other centers had values at the assay's detection limits (0.03 pg/mL and 10 pg/mL in two cases) and were similarly excluded.

The final cohort comprised 185 participants, with a mean (standard deviation [SD]) age of 71.5 (5.9) years; 53% were female, and 66.5% were CSF‐defined Aβ‐positive. Diagnostic groups included 118 ad, 50 SND, 12 FTD (3 Aβ‐positive, 25%), and 5 DLB (2 Aβ‐positive, 40%). Compared to Aβ‐negative participants, Aβ‐positive individuals had significantly lower Mini‐Mental State Examination (MMSE) scores (23.4 ± 4.3 vs. 24.8 ± 3.8; *p* = 0.054) and lower body mass index (BMI; 26.0 ± 4.5 vs. 28.6 ± 3.9 kg/m^2^; *p* < 0.001). No significant differences were observed in age, age at onset, sex distribution, or estimated glomerular filtration rate. As expected, Aβ‐positive participants had markedly lower CSF Aβ1–42 concentrations and higher CSF p‐tau181 and plasma p‐tau217 levels (all *p* < 0.001). When comparing participants recruited at HCB (*n* = 84) with those from the other participating centers (*n* = 101), the HCB group showed a lower BMI (25.9 ± 4.4 vs. 27.6 ± 4.4 kg/m^2^; *p* = 0.016). No differences were observed in age, age at onset, sex distribution, MMSE, CSF Aβ1–42, CSF p‐tau181, or plasma p‐tau217. Full demographic, clinical, and biomarker data, stratified both by Aβ status and by recruitment center, are summarized in Table 1.

**TABLE 1:** Comparison of demographic, clinical, and biomarker characteristics stratified by Aβ status and by recruitment center.

	Total (*n* = 185)	Aβ − (*n* = 62)	Aβ + (*n* = 123)	*p* ^1^	HCB (*n* = 84)	Other centers (*n* = 101)	*p* ^2^
Age (years)	71.50 (5.95)	70.63 (6.85)	71.94 (5.41)	0.39	71.44 (5.71)	71.54 (6.17)	0.93
Age at onset	69.37 (6.12)	68.50 (6.89)	69.80 (5.63)	0.32	69.10 (5.73)	69.60 (6.42)	0.65
Sex (female) *n* (%)	97 (52.4%)	29 (46.8%)	68 (55.3%)	0.35	38 (45%)	59 (58%)	0.10
MMSE	23.89 (4.15)	24.78 (3.78)	23.39 (4.28)	0.05	24.43 (3.86)	23.60 (4.39)	0.28
BMI (kg/m^2^)	26.88 (4.46)	28.55 (3.88)	25.99 (4.50)	< 0.001	25.94 (4.40)	27.61 (4.39)	0.016
eGFR (mL/min)	78.55 (12.95)	79.75 (13.35)	77.94 (12.75)	0.19	77.55 (12.36)	79.39 (13.43)	0.10
CSF Aβ1‐42 (pg/mL)	671.44 (386.67)	1052.34 (413.00)	479.45 (171.22)	< 0.001	615.82 (332.52)	717.70 (422.58)	0.098
CSF p‐tau181 (pg/mL)	85.41 (67.82)	42.27 (21.62)	107.16 (72.68)	< 0.001	81.40 (64.40)	135.20 (349.90)	0.71
Plasma p‐tau217 (pg/mL)	0.57 (0.48)	0.21 (0.15)	0.76 (0.48)	< 0.001	0.62 (0.48)	0.54 (0.47)	0.13

*Note:* Continuous variables are expressed as mean (standard deviation) and compared using Student's *t*‐tests or Mann–Whitney *U* tests, according to distribution. Categorical variables (sex) are expressed as a number (percentage) and compared using *χ* ^2^ tests. A *p*‐value < 0.05 was considered statistically significant. Missing data affected MMSE (*n* = 31), BMI (*n* = 18), eGFR (*n* = 2) and age at onset (*n* = 5), with no apparent clustering across diagnostic groups. All other variables, including CSF Aβ1–42, CSF p‐tau181, plasma p‐tau217, and sex, had complete data.

Abbreviations: Aβ, amyloid‐β; BMI, body mass index; eGFR, estimated glomerular filtration rate; HCB, Hospital Clínic de Barcelona; MMSE, Mini‐Mental State Examination; SD, standard deviation. *p*‐value^1^ compares Aβ– versus Aβ + groups. *p*‐value^2^ compares the tertiary center (HCB) versus the six remaining centers.

### 3.2 Diagnostic Performance of Plasma p‐tau217 for Amyloid Status

**FIGURE 1:**
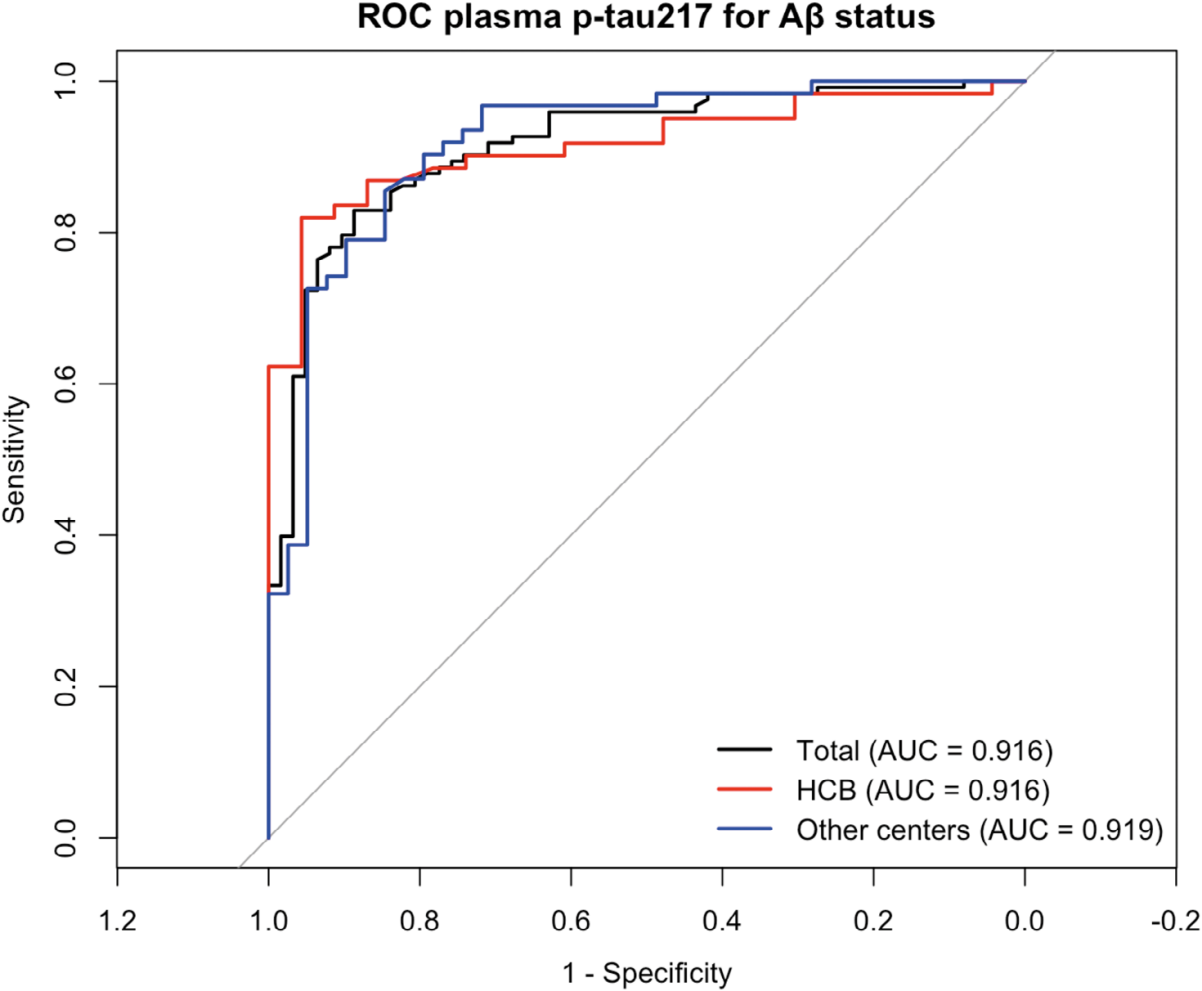
Receiver operating characteristic (ROC) curves of plasma p‐tau217 for identifying CSF‐defined Aβ positivity in the overall cohort (*n* = 185), (HCB, *n* = 84), and the other participating centers (*n* = 101).

We first evaluated the diagnostic accuracy of plasma p‐tau217 for identifying CSF‐defined Aβ positivity in the entire cohort. ROC curve analysis demonstrated excellent discriminative ability, with an AUC of 0.916 (95% CI: 0.875–0.958) (Figure 1). Using Youden's index, the optimal single cut‐off value was 0.336 pg/mL, yielding 82.9% sensitivity, 88.7% specificity, and an overall diagnostic accuracy of 84.9%. Diagnostic performance was highly consistent across centers. The AUC was 0.916 (95% CI: 0.857–0.976) at the tertiary hospital (HCB) and 0.919 (95% CI: 0.862–0.976) across the other centers, with no significant difference by DeLong's test (Figure 1). The optimal cut‐off was 0.360 pg/mL at the tertiary hospital and 0.300 pg/mL when combining the other participating centers. When applying the global cut‐off of 0.336 pg/mL, diagnostic accuracy remained stable across sites: 86.9% at HCB and 83.2% at other centers. This difference was not statistically significant.

To enhance clinical applicability, we implemented a dual‐threshold algorithm that stratified patients into three probability categories for Aβ positivity: low, intermediate, and high. In the lenient approach (targeting 95% sensitivity and specificity), a lower cut‐off of 0.207 pg/mL achieved 95.1% sensitivity and an 86.7% negative predictive value (NPV), identifying 24.3% of participants as low probability of Aβ positivity. An upper cut‐off of 0.397 pg/mL yielded 95.2% specificity and a 96.7% positive predictive value (PPV), assigning 49.7% to the high‐probability group. The remaining 25.9% of participants fell into the intermediate zone. This strategy resulted in an overall diagnostic accuracy of 93.4%.

A stricter approach, targeting 97.5% sensitivity and specificity, applied cut‐offs of 0.146 pg/mL and 0.486 pg/mL. This model achieved a slightly higher overall accuracy (95.3%), with a PPV of 97.4% and an NPV of 89.7%. However, it also increased the proportion of individuals falling into the intermediate zone to 42.7%, thereby reducing the number of cases that could be confidently classified on the basis only of plasma biomarkers. Finally, we tested the robustness of the strict dual‐threshold algorithm by simulating a ±10% variation in p‐tau217 values, accounting for potential analytical or pre‐analytical variability. The model remained highly accurate under a ±10% variation (93.4%). The performance metrics of all models are summarized in Table 2.

**TABLE 2:** Diagnostic performance of plasma p‐tau217 using three dual‐threshold algorithms for predicting CSF‐defined Aβ status.

Algorithm	Lenient (95%)	Strict (97.5%)	Strict (97.5%) with 10% variation margin
Lower cut‐off (pg/mL)	0.207	0.146	0.131–0.161
Higher cut‐off (pg/mL)	0.397	0.486	0.437–0.535
Sensitivity at lower cut‐off (%)	95.1	97.6	95.9
Specificity at higher cut‐off (%)	95.2	96.8	95.2
NPV (%)	86.7	89.7	85.3
PPV (%)	96.7	97.4	96.6
Low‐probability group (*n*, %)	45 (24.3%)	29 (15.7%)	34 (18.4%)
High‐probability group (*n*, %)	92 (49.7%)	77 (41.6%)	88 (47.6%)
Intermediate group (*n*, %)	48 (25.9%)	79 (42.7%)	63 (34.1%)
Further testing spared (*n*, %)	137 (74.1%)	106 (57.3%)	122 (65.9%)
Accuracy (%)	93.4	95.3	93.4

*Note:* The table summarizes the performance of three dual cut‐off strategies to classify individuals into low, intermediate, or high probability of CSF Aβ positivity on the basis of plasma p‐tau217 levels. The lenient model applies thresholds targeting 95% sensitivity and specificity; the strict model applies 97.5% thresholds. A third model assesses the robustness of the strict approach by simulating a ±10% variation in plasma concentrations.

Abbreviations: Aβ, amyloid beta; NPV, negative predictive value; PPV, positive predictive value.

**FIGURE 2:**
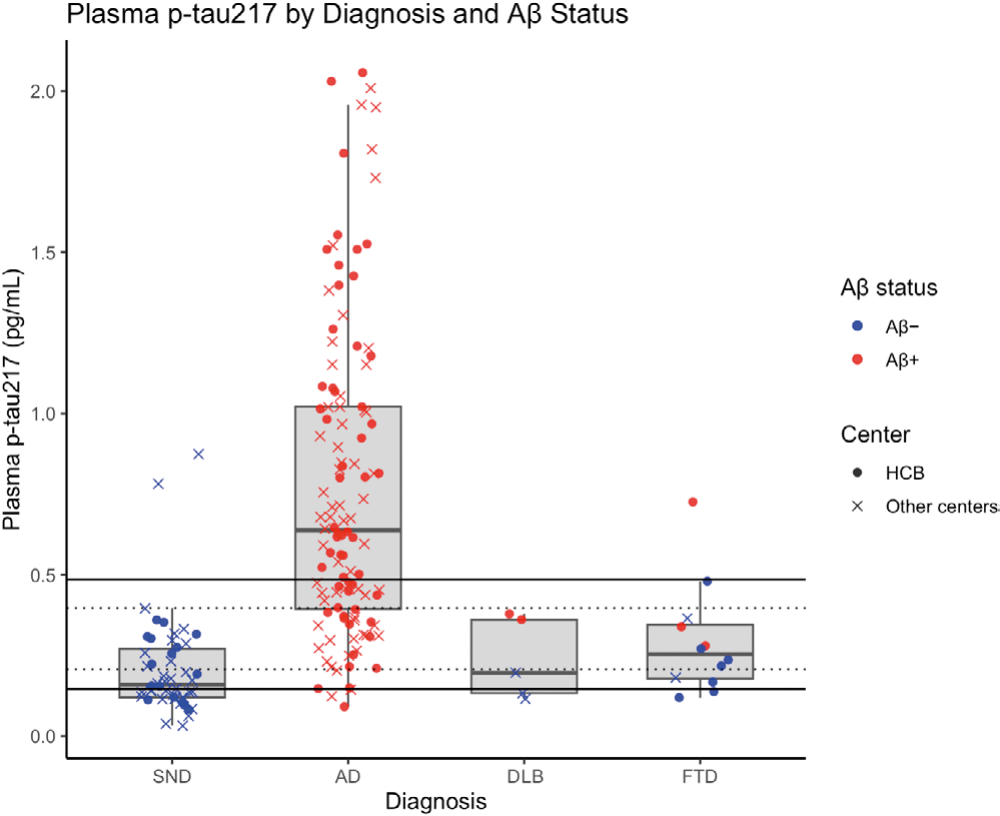
Distribution of plasma p‐tau217 concentrations by clinical diagnosis and Aβ status, with reference lines for lenient and strict dual‐threshold models. Boxplots show plasma p‐tau217 levels across clinical diagnostic groups (SND, AD, DLB, FTD), stratified by CSF‐defined Aβ status (red = Aβ‐positive, blue = Aβ‐negative). Individual data points are overlaid, with circles indicating participants recruited at the tertiary hospital (HCB) and crosses those from the other centers. Boxplots indicate median and interquartile range. The dashed horizontal lines represent the dual‐threshold model based on 95% sensitivity and specificity (lenient approach), whereas the solid lines correspond to the 97.5% sensitivity/specificity model (strict approach). SND, suspected non‐neurodegenerative cognitive impairment; AD, Alzheimer's disease; DLB, dementia with lewy bodies; FTD, frontotemporal dementia; Aβ−/+, amyloid beta negative/positive; p‐tau217, plasma tau phosphorylated at threonine 217.

When comparing the tertiary hospital and other centers, diagnostic accuracy remained consistently high under the dual‐threshold approach. Using the lenient thresholds (0.204/0.397 pg/mL), 29.8% of the tertiary hospital participants and 22.8% of participants from other centers were classified as intermediate. Among the evaluable cases, accuracy was 93.2% at the tertiary hospital and 93.6% at other centers, with no significant difference between groups. With the stricter thresholds (0.146/0.487 pg/mL), accuracy reached 97.8% in the tertiary hospital and 93.3% in other centers. Accuracy did not differ significantly between sites. The distribution of plasma p‐tau217 values and the dual‐threshold classification across centers are shown in Figure 2.

### 3.3 Plasma p‐tau217 Across AT Biomarker Profiles

**FIGURE 3:**
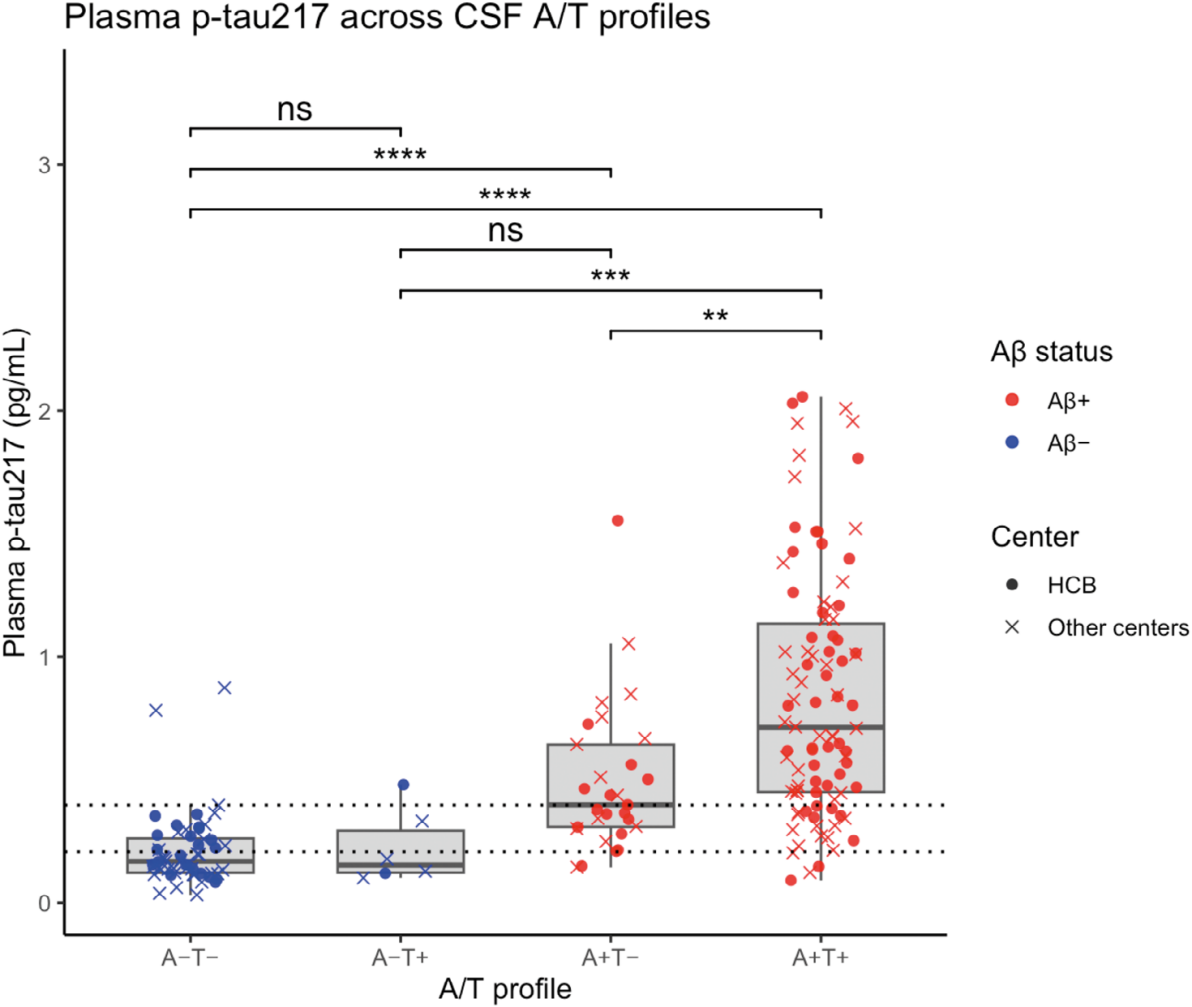
Distribution of plasma p‐tau217 across AT biomarker profiles. Boxplots show plasma p‐tau217 concentrations according to CSF‐defined A/T classification: A − T− (*n* = 56), A − T+ (*n* = 6), A + T− (*n* = 29), and A + T+ (*n* = 94). Pairwise comparisons were conducted using Dunn's test with Bonferroni correction. Asterisks indicate statistical significance: ****p < 0.0001; **p < 0.01; *p < 0.05; ns = not significant. The dashed horizontal lines represent the dual‐threshold model based on 95% sensitivity and specificity (lenient approach), whereas the solid lines correspond to the 97.5% sensitivity/specificity model (strict approach). A, amyloid status; T, tau status; p‐tau217, plasma tau phosphorylated at threonine 217.

To examine the relationship between plasma p‐tau217 levels and AD pathophysiology, we analyzed its distribution across CSF‐defined A/T biomarker profiles. Participants were categorized into four groups: A − T− (*n* = 56), A − T+ (*n* = 6), A + T− (*n* = 29), and A + T+ (*n* = 94). Plasma p‐tau217 concentrations differed significantly across AT profiles (Kruskal–Wallis test, H = 92.79, *p* < 0.001). In post hoc comparisons using Dunn's test with Bonferroni correction, the A + T+ group showed the highest plasma p‐tau217 levels, significantly greater than in A + T− (*p* < 0.05), A − T+ (*p* < 0.01), and A − T− participants (*p* < 0.0001). Levels were also higher in the A + T− group compared with the A − T− group (*p* < 0.0001). In contrast, no significant differences were observed between the A − T+ and A − T− groups or between the A + T− and A − T+ groups (Figure 3).

We also examined how each CSF‐defined A/T profile was distributed across the three diagnostic probability categories derived from the dual‐threshold plasma p‐tau217 algorithms (lenient and strict). Most A + T+ individuals were assigned to the high‐probability group under both thresholds—78.7% with the lenient and 68.1% with the strict one. In contrast, A − T− participants predominantly fell into the low‐probability group with the lenient model (62.5%), whereas under the strict model, they were more often intermediate (55.4%) than low (41.1%). A + T− cases were mainly intermediate with the stricter model (58.6%) but shifted toward the high‐probability category under the lenient model (51.7%). The small A − T+ group showed heterogeneous results, with most individuals assigned to the low or intermediate categories depending on the model (Table 3).

**TABLE 3:** Distribution of AT biomarker profiles across diagnostic probability groups defined by lenient and strict dual‐threshold p‐Tau217 models.

AT profile	Total (*n*)	Lenient model			Strict model		
		Low	Intermediate	High	Low	Intermediate	High
A − T−	56	35 (62.5%)	19 (33.9%)	2 (3.6%)	23 (41.1%)	31 (55.4%)	2 (3.6%)
A − T+	6	4 (66.7%)	1 (16.7%)	1 (16.7%)	3 (50.0%)	3 (50.0%)	0 (0.0%)
A + T−	29	2 (6.9%)	12 (41.4%)	15 (51.7%)	1 (3.4%)	17 (58.6%)	11 (37.9%)
A + T+	94	4 (4.3%)	16 (17.0%)	74 (78.7%)	2 (2.1%)	28 (29.8%)	64 (68.1%)
Total	185	45 (24.3%)	48 (25.9%)	92 (49.7%)	29 (15.7%)	79 (42.7%)	77 (41.6%)

Abbreviations: A, amyloid status; p‐tau217, plasma tau phosphorylated at threonine 217; T, tau status.

Additionally, we evaluated the performance of plasma p‐tau217 for identifying combined amyloid and tau positivity (A + T+). In the whole cohort, the AUC was 0.864 (95% CI: 0.808–0.915), with comparable performance in HCB (AUC 0.884) and the other centers (AUC 0.856) (Supplementary Figure 1).

### 3.4 Impact of Comorbidities on Plasma p‐tau217

We evaluated whether common demographic and clinical variables could influence plasma p‐tau217 concentrations or affect its diagnostic performance for detecting Aβ pathology. In a multivariate linear regression model with age, sex, and Aβ status (*n* = 165), both lower eGFR (standardized *β* = −0.191, *p* = 0.011) and lower BMI (standardized *β* = −0.286, *p* < 0.001) were significantly associated with higher plasma p‐tau217 levels. In contrast, neither age nor sex showed significant associations.

**FIGURE 4:**
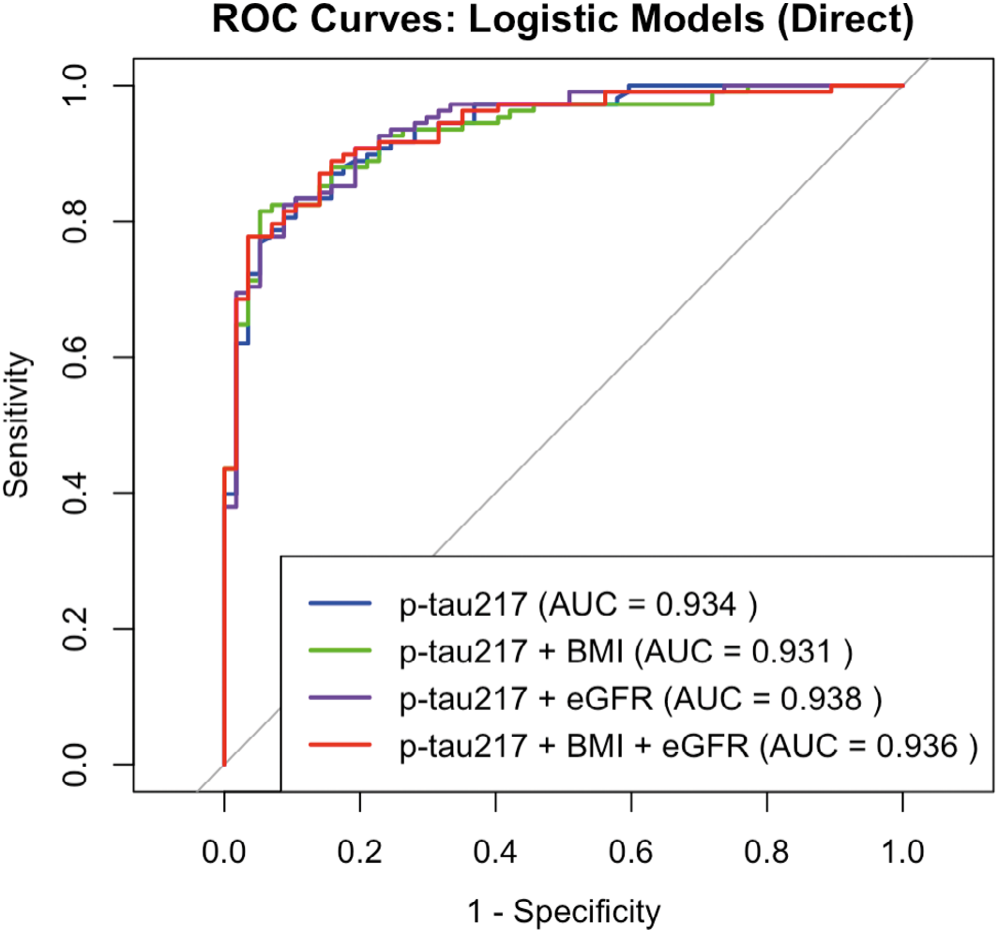
Comparative ROC curves for logistic models predicting CSF‐defined Aβ status. ROC curves compare the diagnostic performance of four logistic regression models: Plasma p‐tau217 alone (blue), adjusted for BMI (green), for eGFR (purple), or for both BMI and eGFR (red).

To assess whether adjusting for these variables improved diagnostic performance, we compared four logistic regression models for predicting CSF‐defined Aβ status: (1) plasma p‐tau217 alone, (2) p‐tau217 + BMI, (3) p‐tau217 + eGFR, and (4) p‐tau217 + BMI + eGFR. All models demonstrated high diagnostic accuracy, with areas under the curve (AUCs) ranging from 0.931 to 0.938. Importantly, none of the adjusted models significantly outperformed the unadjusted p‐tau217 model (all *p* > 0.48, DeLong's test) (Figure 4).

## 4 Discussion

In this multicenter study, we validated the diagnostic performance of plasma p‐tau217 for identifying AD pathology across both tertiary and non‐tertiary clinical settings. Plasma p‐tau217 robustly discriminated AD from non‐AD participants, with highly consistent accuracy across centers, supporting its generalizability in routine clinical practice. The use of an indeterminate range improved interpretability by clearly separating rule‐in and rule‐out cases while flagging those requiring confirmatory testing. Describing the relationship between CSF A/T status and plasma p‐tau217 values helped interpret the three ranges by showing expected biological gradients across AT profiles. Demographic and clinical factors had only a minor influence on performance, indicating that plasma p‐tau217 provides reliable diagnostic information across diverse patient profiles.

Overall, our findings provide empirical support for the 2024 Alzheimer's Association criteria, which allow BBM‐only diagnostic pathways when accuracy exceeds 90% [20]. Plasma p‐tau217 met this threshold in our cohort, demonstrating that fully automatic assays can deliver high‐quality performance across clinical environments. A two‐cut‐off approach may further optimize diagnostic workflows by reserving CSF or PET for indeterminate cases, improving access to biomarker testing, and reducing reliance on invasive or costly procedures. This aligns with the growing demand for scalable verification strategies as anti‐amyloid therapies become increasingly accessible [9, 20, 27].

Our findings are consistent with and extend prior evidence supporting plasma p‐tau217 as a reliable biomarker for biological AD. Previous studies–including our own memory‐clinic cohort and large multicounty evaluations–have consistently reported AUCs in the 0.90–0.96 range, with improved accuracy when dual‐threshold strategies are applied [14, 15, 17, 18, 19, 23, 24, 25, 34]. Importantly, our direct comparison between a tertiary‐memory clinic and six additional centers reinforces the generalizability of standardized thresholds on a centralized automated platform.

A common limitation in the field is reliance on single‐center or research‐enriched cohorts. Our prospective, multicenter design addresses this gap. CSF A/T determinations were performed locally using routine site‐specific cut‐offs, whereas plasma p‐tau217 was analyzed centrally on a fully automated platform under uniform SOPs. Despite the heterogeneity introduced by local CSF procedures, we observed nearly identical AUCs across sites and no significant accuracy differences when applying a single global plasma cut‐off. This supports harmonized adoption of centralized plasma workflows and demonstrates that blood can be collected in smaller or remote centers and shipped to a tertiary facility without compromising diagnostic performance. Such a model improves access, reduces patient travel, and facilitates equitable regional implementation.

Plasma p‐tau217 followed AT biology and aided interpretation of gray‐zone profiles. Most A + T+ fell within the high‐probability range, whereas A − T− clustered in the low‐probability band. A + T− cases were predominantly intermediate with strict thresholds but shifted toward higher probability under lenient criteria–consistent with the view that A + T− represents a transitional stage in which soluble p‐tau rises before extensive tau accumulation. This aligns with longitudinal studies showing that plasma p‐tau217 increases earlier than tau‐PET [16, 17, 35, 36], and with our previous findings [26]. Indeterminate results should be interpreted in a clinical context: confirmation is warranted when certainty is essential, whereas typical presentations near the intermediate‐zone boundaries may not require immediate escalation.

Renal function and body mass index influenced plasma p‐tau217 levels, but adjusting for eGFR or BMI did not meaningfully alter the discrimination of CSF Aβ status. Thus, routine adjustment is unnecessary in memory‐clinic populations [13, 25, 37, 38]. Chronic Kidney Disease (CKD) may expand the intermediate band by elevating p‐tau217 among Aβ‐negative individuals; ratios such as p‐tau217:Aβ42 may mitigate this effect and could be used for triage in primary‐care settings [27, 38]. Overall, these factors have a limited impact, but confirmatory testing should be considered when values lie near diagnostic thresholds in patients with markedly abnormal eGFR or BMI.

A practical observation was the impact of pre‐analytical variability: 12.7% of samples were excluded because of site‐specific anomalies characterized by uniformly elevated p‐tau217 values. Likely contributors include deviations in centrifugation conditions, tube type, and delays in processing. Harmonized SOPs–including standardized time‐to‐centrifugation, temperature logging, and aliquoting–together with inter‐site quality control, will be essential for reliable implementation [21, 22, 33]. Importantly, once compromised samples were excluded, performance remained high and reproducible across centers.

Strengths of this study include the use of a real‐world multicenter cohort, an explicit assessment of between‐site reproducibility, centralized automated analysis, and validation of dual‐threshold algorithms, including robustness testing. Limitations include the absence of amyloid‐PET and tau‐PET, lack of neuropathological confirmation, limited non‐AD sample size, and a predominantly European memory‐clinic population, which may limit generalizability. Plasma samples were analyzed in batches rather than in real time, although available evidence suggests minimal impact [31]. The use of locally established CSF cut‐offs may introduce variability, but plasma assay performance remained stable across centers.

Taken together, plasma p‐tau217 measured on a fully automated platform and interpreted using dual thresholds can serve as a front‐line diagnostic tool for biological AD in routine memory clinics. This approach reduces the need for CSF or PET, preserves accuracy, and improves equity by enabling decentralized sample collection and central analysis. Broader implementation will require harmonized pre‐analytical procedures, coordinated quality control, and alignment with updated diagnostic frameworks supporting two‐cutoff models. Future research should extend this strategy to primary care, cost‐effectiveness, tau‐PET–anchored staging thresholds, and more diverse populations.

## Author Contributions

Miquel Massons, Nuria Guillen, Mircea Balasa, and Albert Lladó designed and conceptualized the study. All authors contributed to data acquisition and recruitment of participants. Miquel Massons, Nuria Guillen, Adrià Tort‐Merino, Agnès Pérez‐Millan, and Albert Lladó analyzed and interpreted the data. Miquel Massons and Nuria Guillen drafted the initial manuscript. Mircea Balasa and Albert Lladó supervised the study and critically revised the manuscript for important intellectual content. All authors read and approved the final version of the manuscript.

## Funding

This study was funded by the Beca Fundació Societat Catalana de Neurologia, Generalitat de Catalunya, AGAUR (SGR 2021–01126) and CERCA Programme. Neus Falgàs was recipient of Juan Rodés contract JR22/00014. Diana Esteller received funding from a Rio Hortega grant (CM23/00137).

## Conflicts of Interest

The authors declare no conflicts of interest.

## Supporting information


**Table 1** Site‐specific cerebrospinal fluid (CSF) biomarker cut‐offs used across participating centers.

**Figure 1** Receiver operating characteristic (ROC) curves of plasma p‐tau217 for identifying CSF‐defined combined amyloid and tau positivity (A + T+) in the overall cohort (*n* = 185), HCB (*n* = 84), and the other participating centers (*n* = 101).

## Data Availability

The data that support the findings of this study are available on request from the corresponding author. The data are not publicly available due to privacy or ethical restrictions.
