# Supplementary material for: Predictive Ability of Plasma p‐tau217 for β‐Amyloid Status: A Prospective Multicenter Study
Source: Ann Clin Transl Neurol. 2026 Apr 13:10.1002/acn3.70387. Online ahead of print. doi: 10.1002/acn3.70387 (PMC13394052; doi:10.1002/acn3.70387)
Supplement: Supplementary file 1 — Table 1 Site‐specific cerebrospinal fluid (CSF) biomarker cut‐offs used across participating centers. [file ACN3-9999-0-s001.docx]

**Supplementary Material for: Predictive ability of plasma p-tau217 for β-amyloid status: a prospective multicenter study**

**Supplementary Figure 1. Receiver operating characteristic (ROC) curves of plasma p-tau217 for identifying CSF-defined combined amyloid and tau positivity (A+T+) in the overall cohort (n = 185), HCB (n = 84), and the other participating centers (n = 101).**

| **Center** | **Aβ42 cutoff (pg/mL)** | **Aβ42/40 ratio cutoff** | **t-tau cutoff (pg/mL)** | **p-tau181 cutoff (pg/mL)** |
| --- | --- | --- | --- | --- |
| Hospital Clínic de Barcelona (HCB) | > 600 | > 0.07 | < 385 | < 65 |
| Hospital Universitari Santa Maria de Lleida | > 600 | > 0.069 | < 400 | < 56 |
| Hospital de la Santa Creu de Vic* | > 600 | > 0.07 | < 385 | < 65 |
| Hospital Asil de Granollers* | > 600 | > 0.07 | < 385 | < 65 |
| Hospital Moisès Broggi – Consorci Sanitari Integral | > 599 | > 0.07 | < 392 | < 56.5 |
| Hospital de Figueres | > 599 | > 0.069 | < 404 | < 56.5 |
| Hospital Sant Jaume de Calella | > 750 | > 0.062 | < 522 | < 69.85 |

**Supplementary Table 1. Site-specific cerebrospinal fluid (CSF) biomarker cut-offs used across participating centers.**

Aβ42, Aβ40, total tau (t-tau), and phosphorylated tau at threonine 181 (p-tau181) were quantified using fully automated chemiluminescent enzyme immunoassays (CLEIA) on the Lumipulse platform (Fujirebio). Centers without on-site Lumipulse instrumentation sent samples to the tertiary hospital (HCB) for analysis. Amyloid-positive (Aβ+) and tau-positive (T+) status were defined according to these site-specific cut-offs, as referenced in the main manuscript.

*CSF are analyzed at HCB using the same cut-offs.
