# Supplementary figures and images for: Predictive Ability of Plasma p‐tau217 for β‐Amyloid Status: A Prospective Multicenter Study
Source: Ann Clin Transl Neurol. 2026 Apr 13:10.1002/acn3.70387. Online ahead of print. doi: 10.1002/acn3.70387 (PMC13394052; doi:10.1002/acn3.70387)

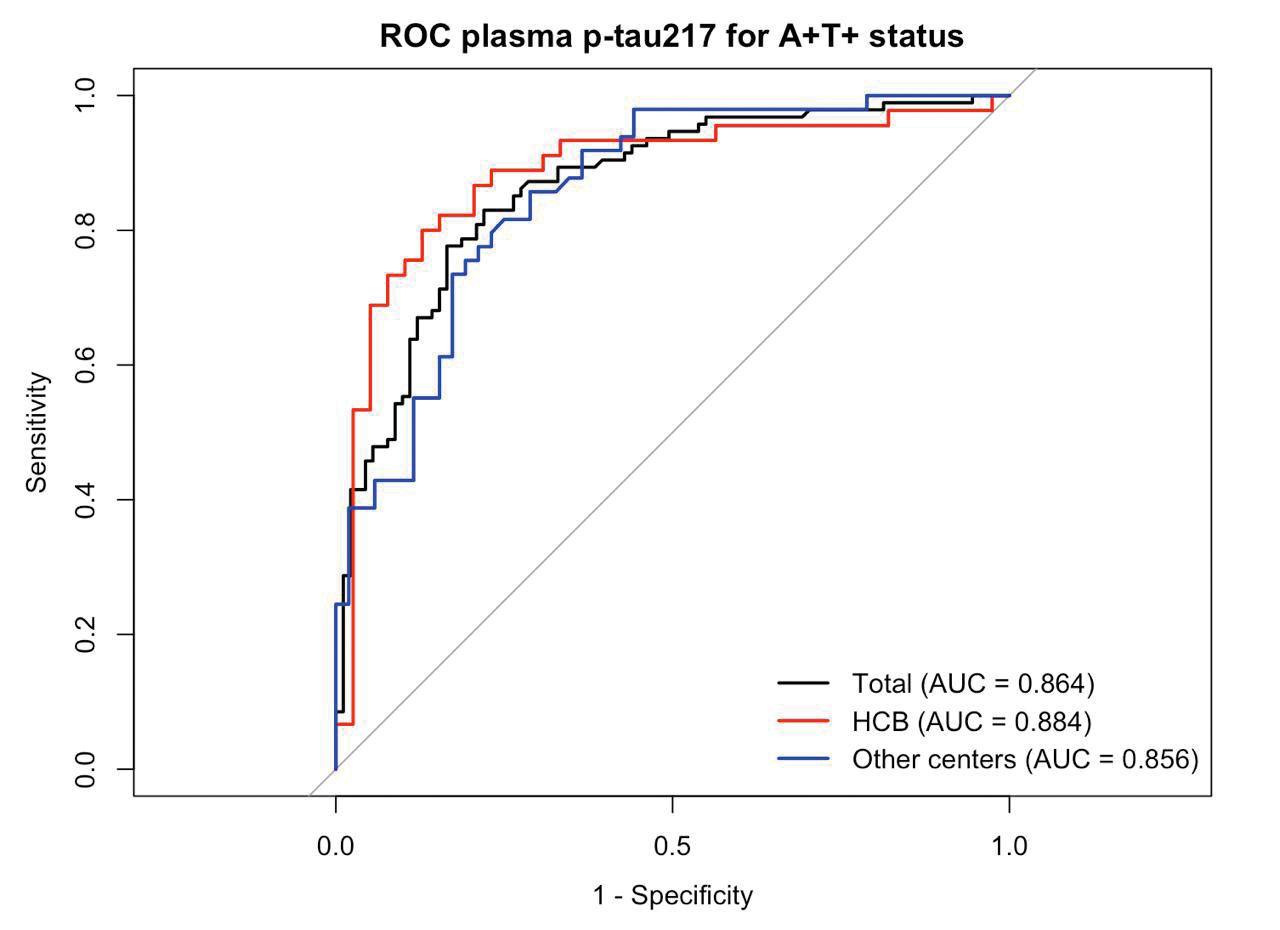

Supplement: Supplementary file 2 — Figure 1 Receiver operating characteristic (ROC) curves of plasma p‐tau217 for identifying CSF‐defined combined amyloid and tau positivity (A + T+) in the overall cohort (n = 185), HCB (n = 84), and the other participating centers (n = 101). [file ACN3-9999-0-s002.png]
